# Online Lifestyle Modification Intervention: Survey of Primary Care Providers’ Attitudes and Views

**DOI:** 10.2196/jmir.8616

**Published:** 2018-06-08

**Authors:** Reem M Hanna, Gary Fischer, Molly B Conroy, Cindy Bryce, Rachel Hess, Kathleen McTigue

**Affiliations:** ^1^ Division of Hospital Medicine University of Colorado School of Medicine Aurora, CO United States; ^2^ Division of General Internal Medicine University of Pittsburgh Pittsburgh, PA United States; ^3^ Division of General Internal Medicine University of Utah School of Medicine Salt Lake City, UT United States; ^4^ Graduate School of Public Health University of Pittsburgh Pittsburgh, PA United States; ^5^ Department of Population Health Science University of Utah Health Sciences Salt Lake City, UT United States

**Keywords:** online intervention, obesity, health information technology, referral model

## Abstract

**Background:**

Online tools are a convenient and effective method of delivering lifestyle interventions to obese adult primary care patients. A referral model allows physicians to efficiently direct their patients to the intervention during a primary care visit. However, little is known of physicians’ perspectives and utilization of the referral model for an online lifestyle modification intervention.

**Objective:**

The aim was to evaluate the response of primary care providers (PCPs) to a referral model for implementing a year-long online intervention for weight loss to obese adult patients.

**Methods:**

The PCPs at six primary care clinics were asked to refer adult obese patients to a year-long online lifestyle intervention providing self-management support for weight loss. Following the 1-year intervention, all providers at the participating practices were surveyed regarding their views of the program. Respondents completed survey items assessing their attitudes regarding the 1-year intensive weight loss intervention and identifying resources they would find helpful for assisting patients with weight loss. Referring physicians were asked about their level of satisfaction with implementing the counseling services using standard electronic health record referral processes. Attitudes toward obesity counseling among referring and nonreferring providers were compared. Impressions of how smoothly the referral model of obesity treatment integrated with the clinical workflow were also quantified.

**Results:**

Of the 67 providers who completed the surveys, nonreferring providers (n=17) were more likely to prefer counseling themselves (*P*=.04) and to report having sufficient time to do so (*P*=.03) than referring providers (n=50) were. Nonreferring providers were more likely to report that their patients lacked computer skills (76%, 13/17 vs 34%, 17/50) or had less access to the Internet (65%, 11/17 vs 32%, 16/50).

**Conclusions:**

Understanding providers’ views and barriers regarding the integration of online tools will facilitate widespread implementation of an online lifestyle modification intervention.

## Introduction

To improve patients’ long-term health and to decrease health care costs, health care providers must play a pivotal role in addressing obesity. The US Preventive Services Task Force recommends screening all adults for obesity. For patients with obesity—a body mass index (BMI) of 30 kg/m^2^ or higher—clinicians should offer or refer patients to a comprehensive behavioral intervention [1,2]. Although primary care providers (PCPs) are aware of the importance of addressing obesity in their patient population, counseling in the primary care setting remains uncommon [3]. In one study, not even a third (29%) of obese adults had a recorded diagnosis of obesity, and only 18% had received counseling for weight reduction, whereas 21% had received advice on physical activity and 25% had received advice on diet [4].

Numerous barriers account for these shortcomings, including the absence of practice tools, lack of training in behavior modification strategies, prohibitive costs, and time constraints [3]. Guidelines recommend using a trained interventionist, such as a registered dietitian, psychologist, exercise specialist, health counselor, or other health professional who adheres to formal protocols in weight management, to provide a comprehensive lifestyle modification intervention that is high intensity, with at least 14 sessions over a 6-month timeframe [5]. Unfortunately, many physicians lack the training or clinical infrastructure to comply with these recommendations.

Health information technology is considered a promising avenue for addressing gaps in care and providing patient-centered, yet affordable, health care services [6]. Online tools are considered a convenient and effective option for delivering lifestyle interventions [7]. However, studies on providers’ perspectives and use of online lifestyle interventions are limited. Providers may not recommend online tools for their patients because they are not familiar with them or may be unconvinced of their efficacy or safety [8]. To improve widespread implementation of Internet tools, such tools must be acceptable to providers and smoothly integrated into their practice. The tools must also be easily implemented in a wide variety of settings at an affordable cost and be sustainable over time [9]. Integration of a health information technology intervention into practice must also maintain workflow and minimize disruption [10].

To enhance the adoption of the clinical intervention, careful implementation is essential for executing the program and achieving accurate results. A referral model, in which the physician initiates a referral for an online weight-loss intervention during the primary care visit, is one approach for integrating evidence-based behavioral care into practice. Physician referrals have been associated with positive outcomes in weight management, such as improved patient attendance in consultation sessions and better completion rates of weight management programs relative to self-referred patients [11]. The goal of this study was to evaluate physician response to a referral model for implementing a year-long online intervention providing self-management support for weight loss to obese adult primary care patients.

## Methods

### Parent Study

As part of a randomized controlled trial, three online lifestyle interventions were implemented in six primary care clinics and compared among 373 patients over 1 year of follow-up [12]. The clinics represented a range of practice settings in Western Pennsylvania, USA, including academic, private, rural, and urban. All the practices were part of the University of Pittsburgh Medical Center health system, which is an integrated health enterprise that is headquartered in Pittsburgh, PA. The clinical sites serve a patient population that is primarily white or African American, which is consistent with the region’s racial and ethnic distribution. A study investigator met with clinicians at each of the participating sites before the intervention to explain the structure and goals of the intervention and evaluation process. The PCPs were asked to refer patients who met the inclusion criteria: BMI of 30 kg/m^2^ or higher, aged 21 to 75 years, and receiving primary care at one of the participating practices. Patients were excluded if they had a myocardial infarction within the last 3 months or their PCP felt that a low-fat diet and moderately intense unsupervised physical activity would be unsafe. Additional exclusion criteria included having a health condition that was likely to impact body weight assessment (eg, severe congestive heart failure or ascites) or influence body weight (eg, cancer requiring treatment in the past year except for nonmelanomatous skin cancer), regular use of prescription medication that is likely to influence body weight, current pregnancy or a plan for pregnancy during the study, an inability to learn adequately from English language audio-taped materials, a perceived lack of basic computer or Internet skills, or a perceived lack of high-speed Internet access. A PCP referral was required for enrollment and only one individual per household could be enrolled.

Physicians used the practice’s standard electronic health record (EHR)-based referral and consultant feedback systems. To facilitate enrollment, the EHR system prompted clinicians to consider referring patients who met the inclusion criteria of obesity (BMI ≥30 kg/m^2^) [13]. While the PCPs provided a referral, the online coach and automated curriculum provided the comprehensive counseling to each patient. The PCPs received feedback on the weight change of each participant over the course of the year via one-page automated graphic reports of weight and behavior change, printed on paper and annotated with adherence data. This approach is similar to the use of paper consultant feedback letters. Referring PCPs were recommended to provide patients enrolled in the intervention with feedback and encouragement at their routine appointment. Institutional review board approval was obtained for this study from the University of Pittsburgh (PRO09080118).

### Primary Care Provider Attitude Survey

The PCPs at the six participating primary care practices were surveyed regarding their views of the program following the 1-year intervention. The survey was anonymous. All providers at the participating practices were contacted by email with survey links, and paper surveys were distributed at practice meetings and resident seminars. Nonresponders received electronic reminder prompts on multiple occasions. Responders could only complete the survey once and were asked to indicate whether or not they referred patients to the intervention.

The survey included questions about the providers’ attitudes toward obesity counseling using a five-point Likert scale anchored at strongly disagree (0) and strongly agree (4). Referring providers were asked whether the EHR referral approach to enrollment and the processes for providing feedback on their patients’ progress with lifestyle change integrated smoothly with their clinical workflow. They were also asked whether they provided their referred patients with feedback on their progress toward lifestyle change. All respondents were also asked about their preferences regarding potential clinical resources for maintaining weight loss.

### Data Analysis

The PCPs were divided into “referring” and “nonreferring” categories based on whether or not they referred at least one patient to the study. Cross-sectional analyses examined sample demographics, compared attitudes toward obesity counseling, and quantified impressions of how smoothly the referral model of obesity treatment integrated with clinical workflow between referring and nonreferring providers. Data were summarized with descriptive statistics. Chi-square tests were used to examine the relationship between referral categories and providers’ attitudes or referring status. Descriptive statistics including means and standard deviation were used. All analyses used Stata 11.1.

## Results

Of 185 providers in the six participating primary care practices, 67 (36.2%) completed the provider surveys (Table 1). Among respondents, 99% (66/67) were physicians and 46% (31/67) were females. The majority were white (72%, 48/67) or Asian (22%, 15/67); 4% (3/67) were Hispanic. Fifty PCPs (75%) had referred at least one patient to the program. When comparing referring providers with nonreferring providers, the two groups did not differ in sex or race/ethnicity, but did vary in training status—71% (12/17) of the nonreferring providers and 12% (6/50) of referring providers were resident physicians (*P*<.001). Among referring providers, most agreed that the referral approach to enrollment (94%, 47/50) and the process of providing 1-year follow-up reports on lifestyle progress (80%, 40/50) integrated smoothly with their normal workflow (Table 2). However, only approximately half of referring providers (52%, 26/52) reported that they typically provided their patients with feedback regarding their efforts to change their lifestyle or body weight throughout the intervention.

Referring and nonreferring providers differed in their counseling preferences (*P*=.04) and perception of whether a clinical encounter provides sufficient time to counsel patients on lifestyle decisions (*P*=.03; Table 2). For example, compared with referring providers, nonreferring providers more often reported a preference for counseling on healthy eating and exercise patterns themselves rather than referring for counseling (somewhat agree: 8%, 4/50; strongly agree: 0%, 0/50) versus 24% (4/17) and 6% (1/17), respectively. Nonreferring providers were more likely to report sufficient time during clinic visits to counsel patients adequately on diet, physical activity, and obesity compared to the referring providers (*P*=.03). Overall, 29% (5/17) of nonreferring providers agreed (somewhat or strongly) that time was sufficient, whereas 8% (4/50) of referring providers agreed (somewhat or strongly). Both referring and nonreferring providers reported that they believed their patients would benefit from advice to lose weight through lifestyle changes even though 18% (12/67) of responders somewhat agreed and 1% (1/67) strongly agreed that their patients were generally not interested in receiving counseling for diet, physical activity, or weight loss. In addition, 19% (13/67) reported that obesity should be managed outside the clinical setting. Nonreferring providers more often reported (somewhat or strongly) that their patients were generally not interested in using Internet-based lifestyle counseling (*P*=.01). Referring and nonreferring providers also differed in whether they raised concerns about their patients’ computer skills (*P*<.001) or Internet access (*P*=.04).

**Table 1:** Demographics of provider respondents (referring providers vs nonreferring providers).

Characteristic			Referring providers (n=50), n (%)	Nonreferring providers (n=17), n (%)		Total (N=67), n (%)		*P* value
Gender (female)			26 (52)	5 (29)		31 (46)		.11
Ethnicity (Latino)			2 (4)	1 (6)		3 (4)		.75
**Race**								.10
	White	38 (76)		10 (59)	48 (72)			
	Black	0 (0)		1 (6)	1 (1)			
	Asian	11 (22)		4 (24)	15 (22)			
	Other	1 (2)		2 (12)	3 (4)			
**Professional status**								<.001
	Resident	6 (12)		12 (71)	18 (27)			
	Fellow	1 (2)		2 (12)	3 (5)			
	Attending	43 (86)		2 (12)	45 (67)			
	Nurse practitioner	0 (0)		1 (6)	1 (1)			

**Table 2:** Attitudes toward obesity counseling among referring and nonreferring providers. N/A: not applicable.

Attitudes and category		Referring providers (n=50), n (%)	Nonreferring providers (n=17), n (%)	Total (N=67), n (%)	*P* value
**Report that referral approach to enrollment integrated smoothly^a^**					N/A
	Yes	47 (94)	N/A	N/A	
	No	3 (6)	N/A	N/A	
**Provided feedback to patients regarding their efforts throughout the intervention^a^**					N/A
	Yes	26 (52)	N/A	N/A	
	No	24 (48)	N/A	N/A	
**Prefer to counsel on healthy eating and exercise patterns, without referring for additional counseling**					.04
	Strongly disagree	19 (39)	2 (12)	21 (32)	
	Somewhat disagree	26 (53)	10 (59)	36 (55)	
	Somewhat agree	4 (8)	4 (24)	8 (12)	
	Strongly agree	0 (0)	1 (6)	1 (2)	
**There is typically sufficient time during the appointment to counsel patients adequately on diet, physical activity, and obesity**					.03
	Strongly disagree	31 (63)	5 (29)	36 (55)	
	Somewhat disagree	14 (29)	7 (41)	21 (32)	
	Somewhat agree	3 (6)	5 (29)	8 (12)	
	Strongly agree	1 (2)	0 (0)	1 (2)	
**Most of his/her patients would not benefit from advice to lose weight through lifestyle modification**					.47
	Strongly disagree	10 (59)	31 (63)	41 (62)	
	Somewhat disagree	5 (29)	8 (16)	13 (20)	
	Somewhat agree	2 (12)	6 (12)	8 (12)	
	Strongly agree	0 (0)	4 (8)	4 (6)	
**Patients are generally not interested in receiving counseling for diet, physical activity, and weight loss**					.45
	Strongly disagree	18 (37)	3 (18)	21 (32)	
	Somewhat disagree	22 (45)	10 (59)	32 (48)	
	Somewhat agree	8 (16)	4 (24)	12 (18)	
	Strongly agree	1 (2)	0 (0)	1 (1)	
**Obesity should be managed outside the clinical setting**					.53
	Strongly disagree	18 (37)	3 (18)	21 (32)	
	Somewhat disagree	22 (45)	10 (59)	32 (49)	
	Somewhat agree	6 (12)	3 (18)	9 (14)	
	Strongly agree	3 (6)	1 (6)	4 (6)	
**Report patients are generally not interested in using Internet-based lifestyle counseling**					.01
	Strongly disagree	9 (18)	0 (0)	9 (14)	
	Somewhat disagree	29 (59)	9 (53)	38 (58)	
	Somewhat agree	11 (22)	5 (29)	16 (24)	
	Strongly agree	0 (0)	3 (18)	3 (5)	
**Report patients generally have minimal computer skills**					<.001
	Strongly disagree	7 (14)	1 (6)	8 (12)	
	Somewhat disagree	25 (51)	3 (18)	28 (42)	
	Somewhat agree	17 (35)	9 (53)	26 (39)	
	Strongly agree	0 (0)	4 (24)	4 (6)	
**Report patients likely lack Internet access**					.04
	Strongly disagree	8 (16)	1 (6)	9 (14)	
	Somewhat disagree	25 (51)	5 (29)	30 (45)	
	Somewhat agree	14 (29)	7 (41)	21 (32)	
	Strongly agree	2 (4)	4 (24)	6 (9)	

^a^Only primary care providers that referred patients to the intervention were asked to comment on the noted questions in the survey.

**Table 3:** Physicians’ preferred resources to be offered in clinic to assist patients with weight loss.

Resources		Referring providers (n=50), n (%)	Nonreferring providers (n=17), n (%)	Total (N=67), n (%)	*P* value
**In-person visit with a health educator or coach**					.21
	Yes	36 (73)	15 (88)	51 (77)	
	No	13 (27)	2 (12)	15 (23)	
**Remainder phone calls from clinic staff**					.21
	Yes	36 (73)	15 (88)	51 (77)	
	No	13 (27)	2 (12)	15 (23)	
**Internet/email contact from health educator or coach**					.02
	Yes	40 (82)	9 (53)	49 (74)	
	No	9 (18)	8 (47)	17 (26)	
**Paper list of community resources for healthy lifestyles**					.29
	Yes	33 (67)	9 (53)	42 (64)	
	No	16 (33)	8 (47)	24 (36)	
**Website compiling information on community resources for healthy lifestyles**					.03
	Yes	35 (71)	7 (41)	42 (64)	
	No	14 (29)	10 (59)	24 (36)	
**Clinic-based walking group or exercises classes**					.78
	Yes	24 (49)	9 (53)	33 (50)	
	No	25 (51)	8 (47)	33 (50)	
**Periodic physician visits**					.98
	Yes	20 (41)	7 (41)	27 (41)	
	No	29 (59)	10 (59)	39 (59)	

Nonreferring providers more often agreed that their patients were likely to have minimal computer skills (76%, 13/17 vs 34%, 17/50), and were likely to lack Internet access (65%, 11/17 vs 32%, 16/50).

Most respondents reported that they found it useful to refer patients to a variety of community counseling resources for promoting healthy lifestyles (Table 3). In-person counseling visits with a health educator or coach, reminder phone calls from clinic staff, and online support from a health educator or coach were the most commonly endorsed resources that could be offered to patients. However, a preference for Web-based support—individualized counseling or an online compilation of community resources for healthy lifestyles—was more common among referring (vs nonreferring) providers.

## Discussion

Although primary care physicians are aware of their potentially pivotal role in addressing the prevalence of obesity, weight counseling in the primary care setting remains uncommon. In this study, we found that a standard, EHR-based clinical referral coupled with consultant feedback processes can integrate online self-management support tools with primary care workflow in a manner that is acceptable to PCPs. Providers who referred patients to these resources were more likely to report that they had limited time for counseling compared with other providers. Conversely, a preference for personally delivering healthy lifestyle counseling was less common among referring PCPs, as was a perception that their patients lacked technical interest, skills, or Internet access. Physicians endorsed a range of resources that could assist patients with weight loss, including online options.

Survey responses offered insight into several potential avenues for improving self-management support for obesity. For example, although physicians received feedback of the weight change of each participant, only 53% of the referring providers discussed this with patients and offered feedback regarding their efforts. Fostering more consistent feedback is potentially important because even minimal physician involvement may enhance outcomes of lifestyle modification interventions [13] and continuity of care in the primary care setting provides an opportunity for long-term lifestyle support. Furthermore, education about the obesity literature may be useful. For example, 20% of the providers in our study reported that obesity should be managed outside the clinical setting. Yet, physicians’ direct discussion of patients’ weight status has been associated with a significant weight loss and could be considered a targetable intervention [14]. In addition, 20% felt that most of their patients were generally not interested in receiving counseling on lifestyle modification, a perception that differs from published data on patients’ preferences [15].

Most of the nonreferring providers were resident physicians (71%). Nonreferring providers more frequently preferred to counsel patients themselves rather than refer for counseling, and tended to report having sufficient time during clinic visits to counsel patients adequately on lifestyle modification. This could reflect the fact that resident physicians are allotted longer clinic visits, which provide them with more time to counsel patients. By choosing to counsel patients themselves rather than referring them to an intervention, residents may be more likely to learn the skills to counsel patients on lifestyle modification. Although these skills are essential, such practice might not be sustainable after the completion of their training.

Concerns about Internet access and minimal computer skills were raised by providers, particularly among those who did not refer patients. From our data, we cannot determine whether these responses reflect real or perceived barriers for patients. Broadband access in the US has expanded considerably in recent years, with more than 80% of the population having access to high-speed Internet access [16,17]. Web use among the minority populations has expanded considerably, particularly among African Americans, between 2000 and 2014, with 85% of white, 81% of Hispanic, and 78% of black non-Hispanic respondents reporting Internet use in 2015 [18]. Furthermore, Internet use has increased disproportionately in populations that have historically shown below-average Internet use, including senior citizens, low-income Americans, and rural adults [16,19]. Yet, concerns over a digital divide remain [18,19], and the topic warrants attention whenever online tools are considered for patient care.

Because of the limited response rate (37%), these data may not reflect the full spectrum of PCP opinion regarding the referral model for weight-loss management. However, because 75% of referring doctors responded, their point of view should be adequately represented. In addition, physician response rates for surveys are often low, usually approximately 10 percentage points lower than that of the general population [20]. As noted previously, resident physicians were among the providers who completed the surveys, so study findings might not be applicable to other centers that lack trainees. The study is cross-sectional and providers were surveyed after the intervention. Thus, providers’ attitudes might have changed due to the exposure to the intervention. Confounders could also be contributing to physicians’ perceptions and views. For example, physicians’ BMI has been associated with likelihood of physician initiating a weight-loss conversation [21]. Another limitation of the study is its lack of generalizability to populations without Internet access. In addition, it is not possible to compare attitudes across different practice settings (eg, rural vs urban). Nevertheless, this study has multiple strengths. To our knowledge, this is one of the first studies to evaluate physicians’ views and compliance with a referral model for integrating online self-management support into primary care practice. In addition, this study involved multiple sites of care that represented a wide range of physicians and a diverse patient population.

Obesity has been recognized as one of the driving forces behind rising health care costs [22]. Primary care providers should be at the forefront of tackling the obesity epidemic. Referring patients to an intensive behavioral counseling intervention has been associated with clinically relevant improvements in health [23]. Online lifestyle interventions can provide a convenient and effective method of weight-loss management. Yet, only by understanding PCPs’ views and barriers regarding the integration of online tools with routine preventive health practice will widespread implementation of evidence-based online tools be achieved.

## Abbreviations

BMI: body mass index
EHR: electronic health record
PCP: primary care provider
